# Supplementary material for: Molecular Imaging Reveals a High Degree of Cross-Seeding of Spontaneous Metastases in a Novel Mouse Model of Synchronous Bilateral Breast Cancer
Source: Mol Imaging Biol. 2021 Jul 26;24(1):104–14. doi: 10.1007/s11307-021-01630-z (PMC8760205; doi:10.1007/s11307-021-01630-z)
Supplement: Supplementary file 1 — (DOCX 3157 kb) [file 11307_2021_1630_MOESM1_ESM.docx]

**Electronic Supplementary Material**

Molecular imaging reveals a high degree of cross-seeding of spontaneous metastases in a novel mouse model of synchronous bilateral breast cancer

Journal: Molecular Imaging and Biology

Shirley Liu^1,2^, Nivin N Nyström PhD^1,2^, John J Kelly PhD^1^, Amanda M Hamilton PhD^1^, Yanghao Fu^1,2^, John A Ronald PhD^1,2^

^1^Robarts Research Institute, University of Western Ontario, London, ON, Canada

^2^Department of Medical Biophysics, University of Western Ontario, London, ON, Canada

Correspondence to: John A Ronald, [jronald@robarts.ca](mailto:jronald@robarts.ca), 519-931-5777 x 24391


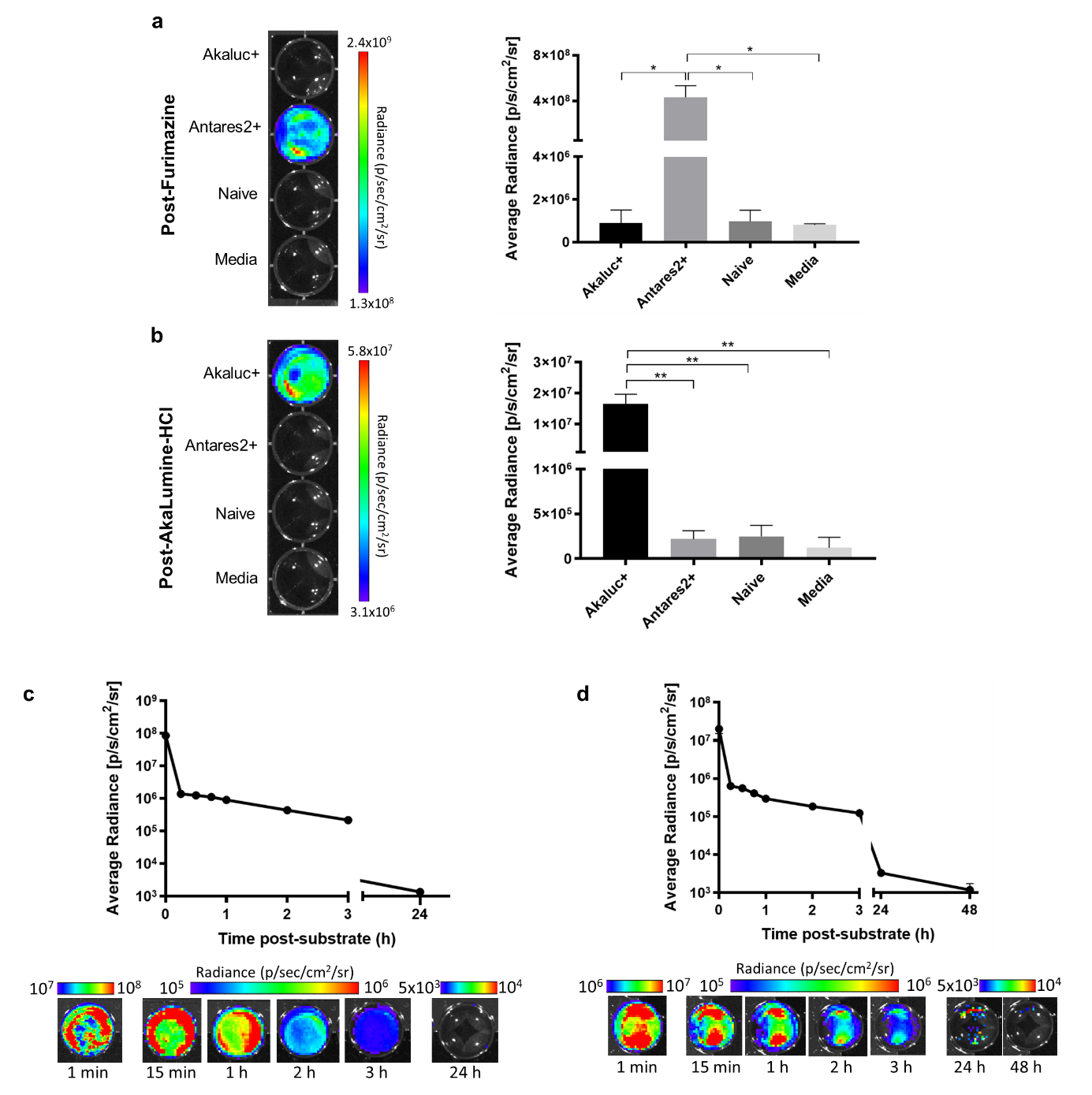
**Supplementary Figure 1.** *In vitro* cross-reactivity and kinetics of Antares2 and Akaluc. Cells were treated with the indicated substrate at 0 hrs, then washed with PBS and incubated in media for subsequent images. Cells were washed with PBS every 24 hours until negligible signal remained: (**a**) Representative well plate and quantification of bioluminescence imaging (BLI) signal of Akaluc-expressing cells, Antares2-expressing cells, naïve cells, and an equivalent volume of media after administration of furimazine (n=3, *p<0.05). (**b**) Representative well plate and quantification of BLI signal of Akaluc-expressing cells, Antares2-expressing cells, naïve cells, and an equivalent volume of media after administration of AkaLumine-HCl (n=3, **p<0.01). (**c**) BLI signal of Antares2-expressing cells over time after administration with furimazine (n=3). (**d**) BLI signal of Akaluc-expressing cells over time after administration with AkaLumine-HCl (n=3). The data are presented as mean ± SEM. Error bars for some data points are smaller than the corresponding symbols.


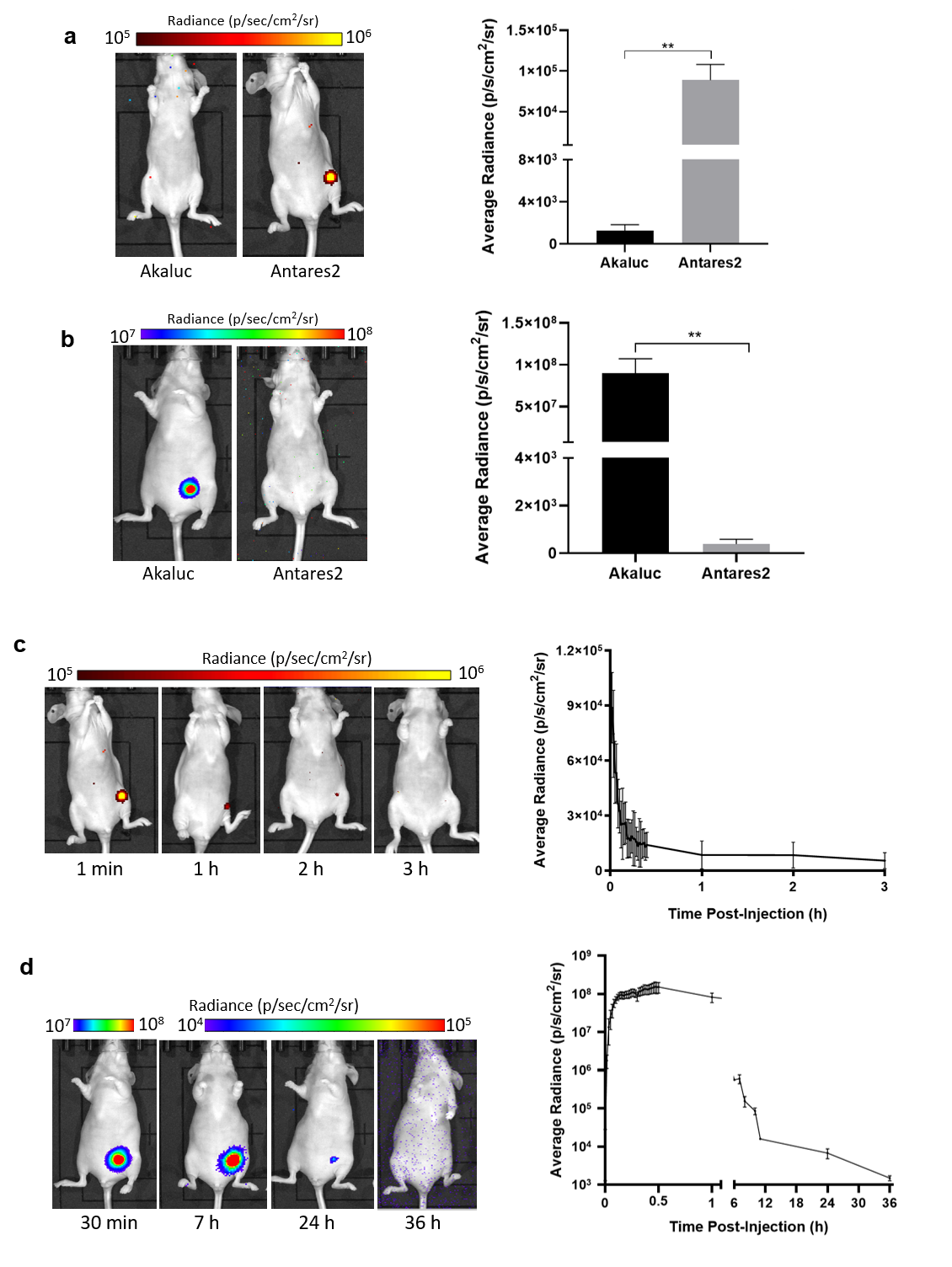


**Supplementary Figure 2.** *In vivo* cross-reactivity and kinetics of Antares2 and Akaluc: (**a**) Representative bioluminescence imaging (BLI) images of a nude mouse bearing an Antares2 mammary fat pad tumor and injected intravenously with furimazine (n=4, **p<0.01). Images were acquired immediately and over time until negligible signal remained. (**b**) Representative images of a nude mouse bearing an Akaluc tumor and injected intraperitoneally with AkaLumine-HCl (n=4, **p<0.01). (**c**) Representative images and quantification of Antares2 BLI signal decay over time. (**d**) Representative images and quantification of Akaluc BLI signal decay over time. The data are presented as mean ± SEM.


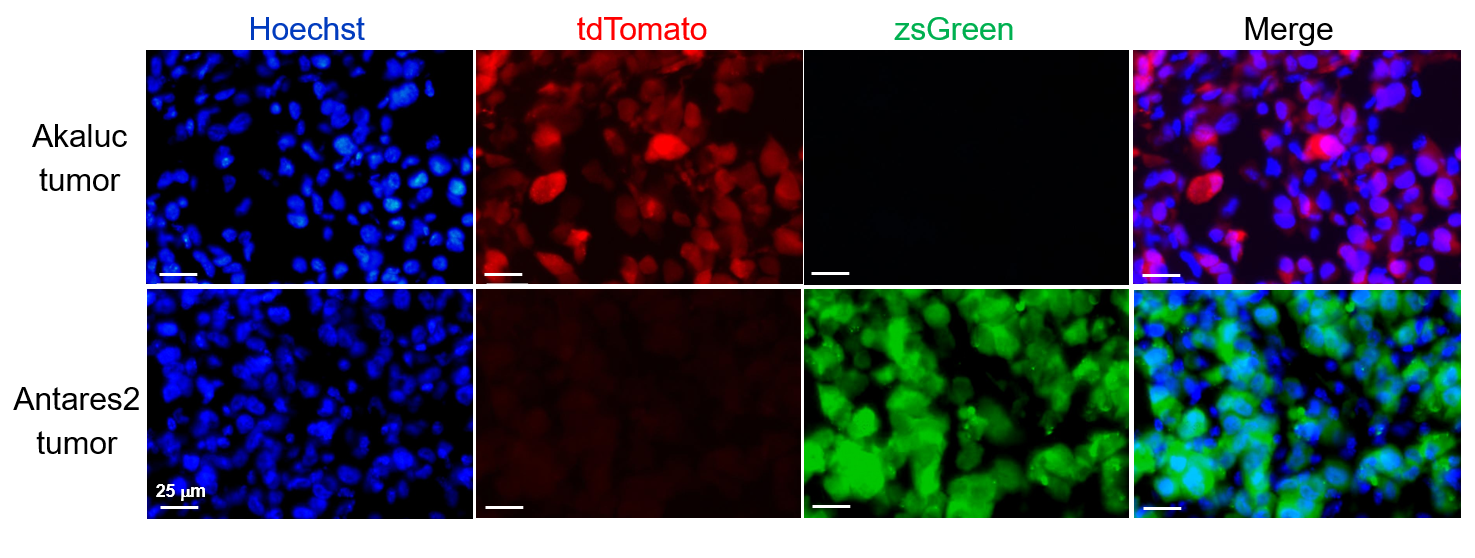


**Supplementary Figure 3.** Fluorescence microscopy images of Akaluc and Antares2 mammary fat pad tumors of mice sacrificed on day 29.


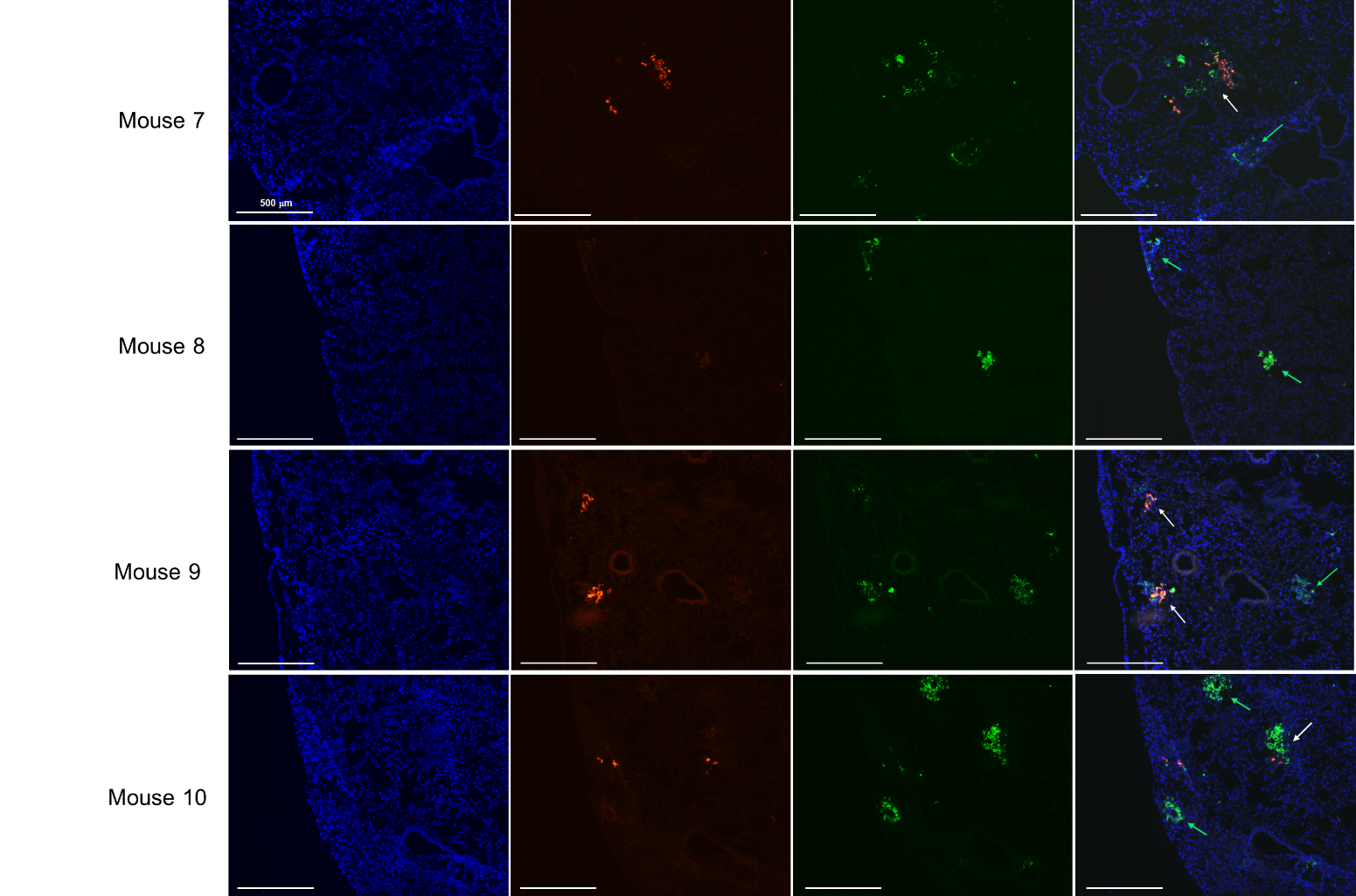


**Supplementary Figure 4.** Fluorescence microscopy images of the lungs of mice sacrificed on day 38. Micrometastases (>200 μm diameter) composed of only zsG-expressing cells and both zsG- and tdT-expressing cells are indicated by green and white arrows, respectively. No micrometastases composed of only tdT-expressing cells were identified in these fields of view.
